# Heterogeneity of Glucocorticoid Resistance in Patients with Bronchial Asthma

**Published:** 2010-09

**Authors:** Yasuhiro Matsumura

**Affiliations:** *Department of Internal Medicine, Akishima Hospital, 1260 Nakagami-cho, Akishima-shi, Tokyo, Japan*

**Keywords:** activator protein-1 (AP-1), bronchial asthma, c-Jun-N-terminal kinase (JNK), glucocorticoid (GC), glucocorticoid receptor (GR), glucocorticoid-resistant (GC-R) asthma, histone deacetylase (HDAC), MAP kinase (MAPK), nuclear factor kappa B (NF-κB)

## Abstract

Bronchial asthma is assumed to be the result of excessive inflammation driven by an aberrant T-helper-2 (Th2) response. Recently, it has begun to be recognized that asthma is a heterogeneous disorder. Glucocorticoids (GCs) are effective treatment for bronchial asthma; however, the inflammation in bronchial asthma cannot always be fully controlled. A recent study demonstrated a new underlying mechanism of glucocorticoid resistance that acts in a Th2-independent manner. Thus, responses to GCs are highly heterogeneous.

## INTRODUCTION

The inflammatory process in asthma is complex and heterogeneous (1, 2). A Th2-mediated disease process plays a role in the clinical manifestations of most bronchial asthma. Interleukin (IL)-4 and -13 induce IgE production, mucus secretion and fibrosis. IL-5 drives eosinophilic inflammation and tissue damage. Epithelium-derived cytokines, including IL-33, facilitate local accumulation and activation of the Th2 response. These in turn cause symptoms.

The Th2 immune process alone is often not enough to explain the persistence of severe asthma. There may be mechanisms by which Th2-like effects are induced in the absence of Th2 cells (3, 4). Emerging data from basic research highlight the involvement of a range of new inflammatory mechanisms, such as Th17 cells and IL-17.

GCs downregulate the mRNAs of various inflammatory cytokines and chemokines while upregulating the mRNAs of molecules that suppress inflammatory cytokines. Despite receiving GC therapy, a small proportion of asthmatic patients have persistent, poorly controlled symptoms. Clinical manifestations of glucocorticoid-resistant (GC-R) asthma largely emphasize the definition of persistence of airway obstruction and failure of FEV1 to improve by 15% over the baseline value after 10 to 14 days of high-dose oral corticosteroids, typically 40 mg of prednisolone daily, when evaluated mainly by reversibility of airflow obstruction (5). A definition referring to the inhalation route must also be taken into account (6).

Many processes involved in inflammation escape modulation by GCs, and resistance to the anti-inflammatory effects of these compounds is mediated by several mechanisms (7-9). The mechanisms of GC-R asthma are not always directly Th2-mediated, and GC responses are highly heterogeneous. The present review discusses important advances in this field based on the recent literature.

## ACTIONS OF GCS

GCs act by binding to the glucocorticoid receptor (GR), which belongs to the family of intracellular ligand-inducible transcription factors termed the steroid/vitamin D/retinoic acid superfamily. These activated GRs translocate to the nucleus and bind to GC response elements (GREs) in the promoter region of steroid-sensitive genes. Nuclear GRs also interact directly or indirectly with coactivator molecules.

Approximately 100 known inflammatory mediators are increased in asthma (10). Increased transcription of small numbers of anti-inflammatory genes does not entirely explain the widespread anti-inflammatory actions of GCs. Furthermore, high concentrations of GCs are required to increase the transcriptions of anti-inflammatory genes (11-13). Switching off inflammatory genes through interactions with transcription factors, such as activator protein-1 (AP-1) and nuclear factor kappa B (NF-κB), may be the major effect of corticosteroids.

GCs act through a combination of direct inhibition of histone acetyltransferase (HAT) activity and recruitment of histone deacetylase (HDAC) to the activated transcriptional complex (14). In asthmatic subjects, there is an increase in HAT activity and a reduction in HDAC activity (15-17).

**Figure 1:**
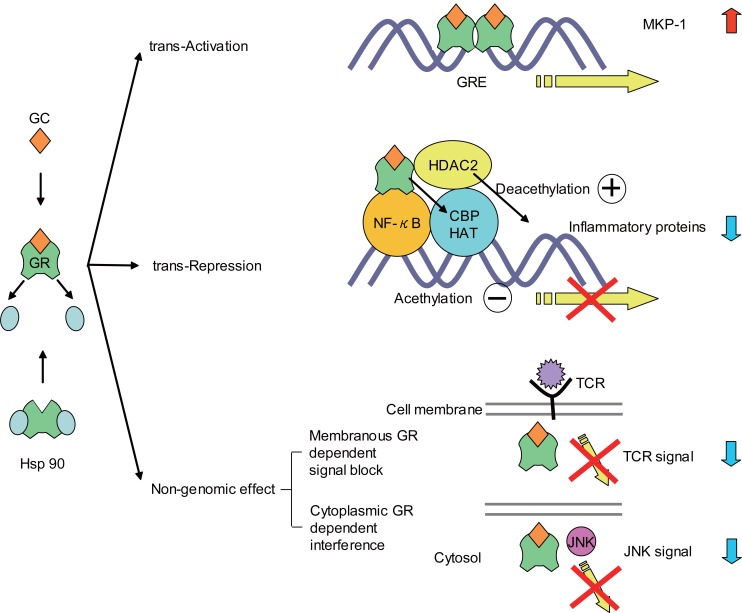
Anti-inflammatory actions of GC. Trans-Activation, GRs bind to GREs and activate genes encoding β2-adrenergic receptors and anti-inflammatory proteins, such as secretory leukoprotease inhibitor (SLPI), MKP-1, IκB-α, and glucocorticoid-induced leucine zipper protein (GILZ). Trans-Repression, GRs inhibit transcription factors such as NF-κB and AP-1. GRs bind to co-activators, such as CBP, and thereby inhibit HAT activity. GRs also recruit HDAC2, leading to suppression of the activated inflammatory genes. Non-genomic effect, GRs modulate signal transduction pathways through physical and functional interaction between the GR and the T-cell receptor (TCR) complex and interference with the JNK signal.

These genomic GC actions do not fully explain the rapid effects of GC. Recent studies have begun to reveal the non-genomic effects of GR, such as membranous GR dependent signal blocking (18, 19) and cytoplasmic GR-dependent interference (20) (Figure 1).

## INTRACELLULAR MECHANISMS OF GC-R ASTHMA

### Protein kinase pathways in GC-R

Intracellular protein kinases are involved in the expression and activation of inflammatory mediators in the airway, through many external inflammatory signals, such as viral and bacterial infections, allergens and cytokines, following binding to transmembrane receptors. MAP kinases (MAPKs), Janus kinases (JAKs), phospho-inositol-3 (PI-3) kinase, inhibitor of κB kinase (IKK) 2 and signal transducers and activators of transcription (STATs) pathways are essential for the inflammatory response. These protein kinases regulate inflammation by activating pro-inflammatory transcription factors such as AP-1 and NF-κB, or via regulation of mRNA stability (21, 22), which in turn results in inflammation.

GCs not only induce MAPK phosphatase 1 (MKP-1), which is an endogenous inhibitor of MAPK genes, but also reduce its degradation (23, 24). MKP-1 inhibits MAPK pathways and thereby inhibits c-Jun-N-terminal kinase (JNK) and to a lesser extent extracellularly regulated kinase (ERK). One effect of these inhibitions is reduced stability of AU-rich element-containing mRNA, which activates inflammation (25).

P38 MAP kinase is more highly activated, possibly as a result of impaired inducibility of MKP-1, in alveolar macrophages in asthmatic patients with a poor response to GCs than in those with a normal response (26). JNK, which is activated by tumor necrosis factor (TNF)-α and other pro-inflammatory cytokines, directly phosphorylates GR at Ser 226 to inhibit GRE binding (27). PI3K may contribute to corticosteroid sensitivity by reducing HDAC activity (28). Therapeutic inhibition of PI3Kδ reportedly restores GC function in oxidative stress-induced GC insensitivity in mice (29) (Table 1).

**Table 1:** Protein kinase pathways involved in GC-R asthma

Impaied inducibility of MKP-1
Activation of p38 MAP kinase
Stabilization of inflammation related AU-rich eliment-containing mRNA
JNK activation
Inhibition of GR binding to DNA (attenuate GR function)
PI3K activation
Reducaction of HDAC activity

Kinase pathways are essential in the expression and activation of inflammatory mediators and in immune cell function. MAPK, JNKs, IKK, and PI-3Ks regulate inflammation either through activation of pro-inflammatory transcription factors such as AP-1 and NF-κB, or through regulation of mRNA half-life. GC resistance is associated with increased activation of protein kinases, which might attenuate GR function or reduce HDAC activity.

GC resistance is associated with increased activation of protein kinases, which might stabilize inflammation, as it relates to mRNA, attenuate GR function or reduce HDAC activity. Targeting specific kinases, that are over-expressed or excessively activated in disease states, could be a useful treatment for GC-R asthma.

### Roles of transcription factors in GC-R asthma

Processes of inflammation in the airways of asthmatics are induced by over-expression of chemokines and cytokines. These mediators are downstream targets for transcription factors, which are proteins that bind to the regulatory sequences of target genes, to increase or decrease the rate of gene transcription. Cross-talk between transcription factors may modulate activation of transcriptional complexes and influence steroid actions.

GRs play a central role in steroid action. GRs are transcription factors that regulate the transcription of steroid-responsive genes, and exert anti-inflammatory activity through functional interactions with other transcription factors, such as AP-1 and NF-κB. Their functions are modified by phosphorylation, which is associated with modulation of ligand binding, nuclear translocation, DNA binding, receptor dimerization, and interactions with transcription factors in general.

GRβ is an alternatively spliced form that binds to DNA but cannot be activated by GC, and has been reported to antagonize the trans-activating activity of GRα. The expression of GRβ is significantly increased in some patients with GC-R asthma and GRβ has been suggested to be involved in glucocorticoid resistance (30-34).

AP-1 expression is enhanced in the asthmatic airway by Th2 cytokines (35). Mononuclear cells of GC-R patients show defective inhibition of AP-1 in response to GCs (36). Failure to suppress JNK phosphorylation, leading to failure to suppress c-Jun phosphorylation, leading to dysregulation of AP-1 in GC-R asthma has been suggested by examination of skin biopsy specimens from a tuberculin-induced model (37) and bronchial biopsy sections from asthmatic patients (38).

NF-κB is a critical transcription factor for the production of many inflammatory cytokines, and is closely related to the pathogenesis of asthma. In patients with bronchial asthma, NF-κB activity is increased in airway epithelial cells, submucosal cells, and macrophages from sputum (39-41). Increased levels of activated p65, phosphorylated inhibitor of NF-κBα (p-IκBα), and IKKβ have been documented in peripheral blood mononuclear cells (PBMC) from subjects with severe uncontrolled asthma (42). An excess of active NF-κB in severe uncontrolled asthma may impair the anti-inflammatory action of GCs. Rhinovirus infection is the one of the main causes of asthma exacerbations, provoking steroid refractoriness. Rhinovirus infection activates NF-κB, which leads to cytokine production and expressions of adhesion molecules (43-45).

The zincfinger transcription factor GATA-3 is essential for expressions of the IL-4, IL-5 and IL-13 genes (46, 47). In T lymphocytes, GATA-3 is localized to the cytoplasm. Upon activation, GATA-3 is phosphorylated by p38 MAP kinase and translocates to the nucleus via the nuclear import protein importin-α. GCs inhibit GATA-3 function through a rapid inhibitory effect on GATA-3 nuclear translocation by preferential binding to the shared nuclear import protein importin-α and also by inhibiting p38 MAP kinase through the induction of MKP-1 (48).

GCs act not only to inhibit inflammation and restore tissue architecture, but also to promote innate immune responses in the airways. GCs thereby induce the expressions of numerous host defense molecules, while suppressing the inflammatory response (49). CCAAT enhancer-binding protein (C/EBP) belongs to the basic region-leucine zipper transcription factor family. Six members of this family have been characterized, to date. The effects of GC on host defense and inflammatory responses are mediated by C/EBPβ (50). This enhanced innate immune response in the airway mucosa is a possible explanation for the ability of GCs to reduce asthma exacerbations in which infectious organisms act as triggers. C/EBP is essential for acute phase protein expressions, whereas NF-κB and AP-1 are more important for inflammatory gene expressions and are GC sensitive.

In airway cells, such as fibroblasts and smooth muscle cells, GC-mediated gene transcription is associated with the formation of a complex between GR and another transcription factor, C/EBPα (51). There is accumulating evidence that a decrease in the level of C/EBPα is responsible for inflammation in airway smooth muscle in asthmatics. The loss of C/EBPα in airway smooth muscle cells in these patients may be essential for the loss of GR function since, in these cells, GR forms a critical complex with C/EBPα that allows key anti-inflammatory mediators to be induced (52).

Recent investigations suggest the participation of interferon regulatory factor-1 (IRF-1) in GC-R. IRF-1 influences the differentiation of Th2 cells. Expression of IRF-1 was increased after viral infections (53). This may explain the reduced steroid responsiveness seen in asthma patients with viral infections (54, 55). IRF-1 is critical for regulation of the transcriptional induction of CD38, which plays a role in airway hyper-responsiveness (AHR) and airway inflammation (56). CD38 expression becomes insensitive to GC action via a mechanism involving up-regulation of the GRβ isoform, thus providing a novel in vitro cellular model for ascertaining how GC resistance develops in primary cells (57).

IRF-1 also promotes GC insensitivity in human airway smooth muscle cells by interfering with GR signaling (58). The inhibition of GR function by IRF-1 involves its interaction with transcriptional co-regulator GR-interacting protein 1 (GRIP-1). Under GC-R conditions, enhanced expression of IRF-1 would deplete GRIP-1 from the GR complex, thereby reducing the transcription of GR-dependent genes such as MKP-1 and promoting the expression of IRF-1-dependent pro-inflammatory genes such as CD38 (59) (Table 2).

**Table 2:** Transcription factors involved in GC-R asthma

GR*β*
Ratio of GR*α* to GR*β* expression correlates with GC-R
Inhibition of GR*α* trans-activation
AP-1
Defective inhibition of AP-1
NF-κB
Excess activation inparis anti-inflammatory action of GCs
C/EBP*α*
Loss of C/EBP*α* critical for loss of GR function
IRF-1
Depleting GRIP-1 results in reduction of MKP-1gene transcription and promotion of CD38 expression, thereby up-regulating GR*β*

Multiple signals mediate activation or inhibition of transcription factors, which modulate inflammatory genes,such as AP-1, NF-κB, STAT, nuclear factor of activated T cell (NF-AT), GATA, and GR. Transcription factors may physically interact with each other and modify GC action. These complicated pathways are underlying factors in inflammation and GC-R asthma.

## TH2-INFLAMMATION IN GC-R ASTHMA

The recruitment of Th2-type T cells, cytokines and chemokines underlie GC-R. In patients with GC-R asthma, a combination of increases in both IL-2 and IL-4 reportedly reduced GR binding affinity in PBMC, and these effects were reversible and blocked by interferon (IFN)-γ (60, 61). A study of bronchoalveolar lavage (BAL) fluid showed significantly greater numbers of cells expressing IL-2 and IL-4 mRNA in GC-R asthmatics as compared with GC-sensitive asthmatics (62). Bronchial biopsy specimens from patients with GC-R asthma revealed over-expressions of IL-2, IL-4 and IL-13 and a reduction in the GR affinity of inflammatory cells (63, 64). The reduction in GR function resulting from IL-2 and IL-4 might be mediated via phosphorylation of the receptor by p38 MAP kinase (65).

TNF-α is reportedly associated with severe and refractory asthma (66) IL-33, which is currently described as a promoter of Th2 immunity and systemic inflammation (67), is expressed at higher levels in airway smooth muscle cells in asthmatics. Dexamethasone (DEX) failed to abrogate TNF-α-induced IL-33 expression (68) (Table 3).

**Table 3:** Cytokine profile involved in GC-R asthma

IL-2, IL-4 and IL-13
Reduction in affinity of GRs
(GRs phosphorylation by p38 MAP kinase)
TNF-*α*, IL-33
Failure to abolish TNF-*α*-induced IL-33 up-regulation
IL-10
Reduction of IL-10 of synthesis
Th1 cells and cytokines
AHR by INF-*γ*/TLR-4-MyD88-dependent mechanism
Th17 cells and IL-17
Activation of transcription factors
Induction of neutrophilic inflammation

Failure to supress the production of inflammatory cytokines and to induce the production of anti-inflammatory cytokines associated GC-R. Cytokines induce immune activation, which leads to attenuated GR function, occasionally via activation of protein kinases.

## OTHER MECHANISMS OF GC-R

There are other GC-R mechanisms independent of Th2-inflammation or that induce Th2-like effects in the absence of Th-2 cells.

GC-R asthma is associated with alterations in Th2/Th1 type cytokine gene expression profiles, i.e., failures to suppress the production of inflammatory cytokines and induce the production of anti-inflammatory cytokines. CD4+ T cells from GC-R asthmatics show a marked reduction in their capacity to synthesize IL-10, which inhibits pro-inflammatory cytokine production, antigen presentation, T cell activation and mast cell (MC) and eosinophil functions, following in vitro stimulation in the presence of DEX, as compared with those from GC-sensitive patients with similar disease severity (69).

Th1 cells and cytokines have also been shown to play critical roles in AHR. Increased Th2 cells in the airways of mice mediate eosinophilic inflammation and AHR that can be suppressed by treatment with GCs. Th1 cells, on the other hand, induce steroid-resistant AHR through an INF-γ/TLR4-MyD88-dependent mechanism after priming of the innate host defense system by lipopolysaccharide (LPS) (70).

Eosinophils are not always the key effector cell in bronchial asthma (71). It was hypothesized that at least two different pathologic subtypes of severe asthma would be present in airway tissue: a “classic” eosinophilic process, and a “pathologically non-classic” process, perhaps associated more strongly with neutrophils (72). It has been suggested that non-eosinophilic asthma is driven by persistent innate immune activation (73). Innate effectors of bronchial asthma can be activated outside of the classical Th2 cell paradigm. Airway epithelial cells orchestrate allergic pulmonary inflammation. Expression of epidermal growth factor receptor (EGFR), a marker of epithelial stress/damage, in asthmatic bronchial epithelium is increased in proportion to disease severity (74), which correlates with IL-8 (75), indicating that EGFR could contribute to sustained neutrophilic inflammation. Neutrophils and their products may play an important role in this inflammation. Subjects with neutrophilic asthma would have increased activation of proteolytic enzymes, such as neutrophil elastase (NE), which indicates a protease/anti-protease imbalance, as compared with other asthma phenotypes (76).

Non-eosinophilic asthma is associated with a poor response to GC (77, 78). Neutrophilic inflammation in acute exacerbations of asthma tends to be resistant to treatment with GCs, because it impairs nuclear recruitment of HDAC2 (17). Patients with mild to moderate asthma who have predominantly neutrophilic airway inflammation are relatively unresponsive to treatment with inhaled corticosteroids (79).

Involvement of Th17 cells was reported in a mouse model of GC-R asthma (80). Th17 cells represent a distinct population of CD4 (+) Th cells that mediate neutrophilic inflammation and are characterized by the production of IL-17, IL-22 and IL-6. Th17 cytokine responses are not sensitive to DEX treatment. Th17 cell-mediated airway inflammation and AHR are steroid resistant, indicating a potential role of Th17 cells in GC-R asthma.

IL-17 is a mediator of neutrophil variant and severe neutrophilic asthma (81). IL-17F is derived from Th17 cells, eosinophils, fibroblasts and venous endothelial cells. IL-17F activates transcriptional factors such as C/EBPβ, C/EBPγ and NF-κB. Thus, IL-17F plays a pro-inflammatory role in asthma. IL-17F is clearly expressed in the airways of asthmatics, and its expression level correlates with disease severity (82) (Table 3).

Amphiregulin up-regulated mucin gene expression in airway epithelial cells and up-regulation of amphiregulin in MCs correlated significantly with the extent of goblet cell hyperplasia in the mucosa of patients with bronchial asthma. Amphiregulin was secreted by human MCs after exposure to antigens via aggregation of FcεRI, resulting in sputum production. Its expression was not inhibited by DEX. This may explain GC treatment being largely ineffective in relation to overproduction of sputum (83).

A study of BAL fluid from subjects with GC-R asthma demonstrated classic macrophage activation and induction of LPS signaling pathways, suggesting a contribution of endotoxin exposure to GC-R asthma (84).

An excess of matrix metalloproteinases (MMPs) may be responsible for structural degradation of tissues, whereas an excess of tissue inhibitor of metalloproteinases (TIMP) may promote excessive tissue repair processes and fibrosis. DEX up-regulates TIMP-1 mRNA in BAL fluid cells from patients with GC-sensitive asthma, but not in cells from those with GC-R asthma. Inability of GC to enhance TIMP-1 production causes a shift in the MMP-9/TIMP-1 ratio in GC-R asthma, potentially promoting proteolytic activity in the airways and (85) possibly resulting in abnormal tissue remodeling in the airways, which could explain the diminished reversible response in patients with GC-R asthma. Eosinophilic asthma is characterized by active MMP-9 without free elastase (76).

External influences such as exposure to cigarette smoke, an oxidative stress, inhibit the anti-inflammatory actions of GC. Clinically, patients with bronchial asthma who smoke have an impaired response to GC therapy as compared to those who do not smoke (86). Smoking increases NF-κB activity, resulting in increased expressions of inflammatory genes such as IL-8, MMP and monocyte chemoattractant protein. Smoking can also inhibit GR function by suppressing GR-associated HDAC2 activity and expression (87, 88).

There has been growing interest in the role of vitamin D in asthma. Local conversion of inactive to active vitamin D alters immune function in the lung (89). In asthma patients, reduced vitamin D levels are associated with impaired lung function, increased AHR and reduced responsiveness to GCs (90). Addition of vitamin D3 and DEX to regulatory T (Treg) cells enhances IL-10 secretion, and administration of vitamin D3 to patients with GC-R asthma reportedly enhances the subsequent IL-10 induction in response to DEX (91). Vitamin D has also been noted to modulate Treg function and IL-10 production, which may increase the therapeutic response to GC in GC-R asthma (92). Thus, a low oral intake of vitamin D, less sun exposure, and engaging in mostly light indoor activities might reduce the GC response.

Elevated body mass index (BMI) is associated with a blunted in vitro response to DEX in overweight and obese patients with asthma. This effect is manifested by reduced induction of MKP-1 expression in response to DEX in both PBMC and BAL cells, and is related to enhanced expression of TNF-α in both peripheral and lung immune cells as BMI increases (93) (Table 4).

**Table 4:** Other mechanisms involved in GC-R asthma

Allergens
Reduction in GR binding affinity
Endotoxin
Macrophage activation
Induction of LPS signaling pathway
Infections
Exacerbation of inflammation (activation of NF-*κ*B)
Neutrophilic inflammation
Inbalance of protease/antiprotease indicated by NE
Impaired nuclear recruitment of HDAC
Amphiregulin
Induces sputum production
MMP-9/TIMP-1 ratio
Abnormal tissue remodeling
Cigatette smoke
Increase of NF-κB activity
Reduced HDAC expression and activity
Vit D
Modulation of Treg function and IL-10
BMI
Reduced MKP-1 induction

Factors that induce asthma attacks or exacerbate asthma activate transcription factors and attenuate GR function, occasionally via kinase pathways, and some also influence HDAC2 activity. Clinical manufestations, such as sputum production and remodeling may be associated with GC-R asthma. Lifestyle and habits should also be taken into the account.

## CONCLUSION

The intracellular mechanism of GC-R asthma may be attributable mostly to reduced GR function resulting from enhanced activations of AP-1 and NF-κB and upstream kinase pathways, or reduced HDAC activity. The intensity of inflammation may explain the very common clinical observation that resistance is relative, and patients often respond to high doses of GCs.

Poor responsiveness to GCs is likely to be multifactorial. Asthma phenotypes, infections and environmental conditions all contribute. Some manifestations reflect a Th2-independent or non-eosinophilic process, possibly indicating that the mechanism of GC-R asthma is not simply Th2-mediated, but rather is heterogeneous.
